# Prevalence of Obesity After Living Kidney Donation and Associated Risk Factors: Cardiovascular and Renal Implications

**DOI:** 10.3390/jcm14186411

**Published:** 2025-09-11

**Authors:** Ana Cunha, Manuela Almeida, Beatriz Gil Braga, Sofia Sousa, José Silvano, Catarina Ribeiro, Sofia Pedroso, La Salete Martins, Jorge Malheiro

**Affiliations:** 1 Department of Nephrology, Unidade Local de Saúde de Santo António (ULSSA), 4099-001 Porto, Portugal; anacunha.nefrologia@chporto.min-saude.pt (A.C.); beatrizgilbraga.nefrologia@chporto.min-saude.pt (B.G.B.); jose.silvano.nefrologia@chporto.min-saude.pt (J.S.); sofiapedroso.nefrologia@chporto.min-saude.pt (S.P.); lasaletemartins.nefrologia@chporto.min-saude.pt (L.S.M.); jmalheiro.nefrologia@chporto.min-saude.pt (J.M.); 2 UMIB—Unit for Multidisciplinary Research in Biomedicine, ICBAS—School of Medicine and Biomedical Sciences, University of Porto, 4050-313 Porto, Portugal; 3 ITR—Laboratory for Integrative and Translational Research in Population Health, 4050-313 Porto, Portugal; 4 Department of Nephrology, Hospital Divino Espírito Santo, 9500-370 Ponta Delgada, Portugal; u14704@chporto.min-saude.pt

**Keywords:** living kidney donor, obesity, cardiovascular risk

## Abstract

**Background:** Living kidney donor (LKD) transplantation contributes to mitigating the organ shortage and some programs now accept donors with borderline criteria, such as obesity. However, the long-term impact of these criteria extension remains unclear. **Methods:** This study retrospectively analyzed 306 LKD from 1998 to 2020 to examine obesity trends, predictors, and impact on cardiovascular risk and kidney function. **Results:** Before donation, 49% of donors were normal weight, 41% were overweight, and 10% were obese. Obese donors were older (50.8 ± 8.8 years, *p* = 0.009) and had higher rates of dyslipidemia and hypertension (41%, *p* < 0.001 for both). Over 9 years, obesity rates were stable (8.8–14.8%). A mixed logistic regression model showed that dyslipidemia (OR 6.1, *p* = 0.042), age (OR 0.9, *p* = 0.005) and body mass index (OR 5.3, *p* < 0.001) were strong predictors of post-donation obesity. Overweight donors showed an increase in obesity rates over time in the McNemar’s paired analysis [14% obesity by year 3 (*p* = 0.001); 12.5% at year 10 (*p* = 0.014)]. Post-donation hypertension was more prevalent in obese donors’ (61.1% vs. 30.4%, *p* = 0.011), though proteinuria and estimated glomerular filtration rate (eGFR) did not differ significantly. **Conclusions:** These findings show that pre-donation overweight, younger age, and dyslipidemia predict post-donation obesity, with hypertension posing added risk for obese donors. There was no impact concerning proteinuria and eGFR. The study underscores the importance of careful donor selection and risk informed counseling.

## 1. Introduction

Chronic kidney disease (CKD) is a growing global health issue, affecting millions of people and posing challenges for healthcare systems worldwide [1]. For patients with end-stage kidney disease (ESKD), kidney transplantation remains the most effective treatment, significantly improving survival and quality of life compared to dialysis [2,3,4]. The demand for kidney transplants far exceeds the supply, leaving many patients on the waiting list for extended periods. In Portugal, the median wait time for a kidney transplant is approximately five years, with an annual mortality rate on waiting list of over 5% [4].

The persistent imbalance between organ supply and demand has driven the expansion of living donor transplantation and, in this context, a growing acceptance of donors with borderline medical criteria—including those who are overweight or mildly obese but otherwise healthy [3,5,6]. This strategy is intended to shorten waiting times and to attenuate the deleterious effects of prolonged dialysis, such as elevated cardiovascular risk and increased mortality. This category of “medically complex living donors” now comprises around 25% of living donor’s kidney transplant (LDKT) programs, with data from the Organ Procurement and Transplantation Network [7] showing a 12% increase in overweight and a 20% increase in obese donors from 1999 to 2011. This trend is expected to continue as obesity is an escalating global pandemic. For example, according to Global Obesity Observatory [8], in 2019, 18% of the adult population in Portugal was obese and 38% was categorized as overweight.

Obesity poses unique challenges in kidney transplantation since it implies an increased risk of hypertension, impaired glucose tolerance, type 2 diabetes mellitus, stroke, coronary heart disease, and overall cardiovascular and general mortality [9,10]. It is also known to cause specific kidney alterations, including effacement of podocyte foot processes, focal segmental glomerulosclerosis, and glomerular hypertrophy, referred to as obesity-related glomerulopathy [11,12]. Furthermore, obesity leads to the progression of CKD irrespective of the underlying etiology [10] through different mechanisms like hyperfiltration, renin–angiotensin–aldosterone system activation, and oxidative stress [11,13,14,15,16,17,18,19].

The long-term safety data regarding living kidney donation among individuals with excessive weight is limited [20]. Existing studies raise concerns about potential risks, including an increased incidence of ESKD [21,22,23,24], cardiovascular complications [25] and overall mortality [25] for obese donors. Despite methodological limitations in those studies [26], these findings have highlighted the importance of informed consent [27] and the need for clear guidelines to protect this higher-risk donor population.

The impact of donor’s obesity on post-donation outcomes remains poorly understood in the existing literature, and even less is known about weight changes after kidney donation. As more transplant programs accept overweight and obese individuals as living kidney donors (LKD), understanding the long-term health implications for this group has become essential.

Hence, our study aims to address this knowledge gap by investigating the prevalence and predictors of obesity among LKD over a 15-year follow-up period; assessing changes in obesity status over time in relation to pre-donation body mass index (BMI); identifying risk factors for post-donation obesity; and examining how obesity influences long-term outcomes, particularly in terms of estimated glomerular filtration rate (eGFR) evolution and cardiovascular health.

## 2. Materials and Methods

### 2.1. Patients

We retrospectively reviewed the clinical data of all adult LKDs submitted to nephrectomy at our center between January 1998 and January 2020 (*n* = 365). Inclusion criteria were LKDs with documented body mass index (BMI) at the time of donation and at one-year post-donation. A total of 59 LKDs with missing weight measurements in the medical records were excluded, leaving a final cohort of 306 LKDs for analysis.

This retrospective observational study was approved by the Institutional Review Board of Unidade Local de Saúde de Santo António, under protocol Ref.: 147-21 (119-DEFI/122-CE, 21 September 2021). The study was conducted in compliance with the Declaration of Helsinki and is reported following the Strengthening the Reporting of Observational Studies in Epidemiology guidelines.

### 2.2. Donor Variables

Following international guidelines [28,29], all candidates underwent the standard living donor evaluation protocol, including eGFR using the Chronic Kidney Disease- Epidemiology Collaboration equation [30] (with measured GFR if borderline), urine protein-to-creatinine and albumin-to-creatinine ratios, renal ultrasound, computed tomography angiography, and cardiovascular assessment (electrocardiogram, with echocardiography if >40 years or in the presence of risk factors, and further work-up if indicated). Additional testing included chest radiography, comprehensive blood work and serologies, oral glucose tolerance testing, blood group typing, pregnancy testing in women of childbearing age, and full immunological assessment. Detailed evaluation and exclusion criteria have already been published [31,32]. Baseline demographic, anthropomorphic, analytical and clinical data were collected.

In our cohort, BMI was assessed at baseline and at follow-up. A BMI ≤ 30 kg/m^2^ was strongly recommended, although carefully selected individuals with BMI > 30 and <35 kg/m^2^ were accepted if otherwise healthy and after exclusion of major comorbidities such as hypertension and diabetes or pre-diabetes. For the classification, we considered normal weight a BMI lower than 25 kg/m^2^, overweight a BMI between 25 and 29.9 kg/m^2^ and obesity a BMI higher than 30 kg/m^2^.

Upon urinary analysis, proteinuria (ProtU) was defined by a random urine protein measurement between 0.15 and 0.5 g/g [28], confirmed by a 24 h urine sample. Donors with confirmed proteinuria exceeding 300 mg/day were excluded from donation.

Hypertension during follow-up was defined as blood pressure ≥ 140/90 mmHg or the initiation of antihypertensive therapy; de novo diabetes mellitus was defined as fasting glucose > 126 mg/dL [33] or by the prescription of antidiabetic agents; and dyslipidemia was defined as low-density lipoprotein cholesterol ≥ 115 mg/dL and/or triglycerides ≥ 150 mg/dL, or the use of lipid-lowering medication to manage these levels.

The earliest date of problem onset was considered in the analysis. The date of nephrectomy was considered the start of follow-up, and all donors were offered lifetime annual follow-up appointments. The median follow-up was 6.59 (3.7–10.2) years.

### 2.3. Outcomes

The primary outcome was the occurrence of obesity until 15 years post-donation, using all available weight measurements from donation evaluation onward. Donors were followed from the nephrectomy date until the first of either death, development of end-stage kidney disease (defined as the need of chronic dialysis or a kidney transplant), last follow-up, or end of study period (31 December 2022). An additional analysis with a mixed logistic model was performed to evaluate the influence of demographic and clinical factors at the time of donation on the development of obesity.

The remaining analyses were limited to a 10-year period due to the smaller number of patients with follow-up beyond 10 years post-donation. The association between obesity and other cardiovascular risk factors—such as hypertension, diabetes mellitus, and dyslipidemia—was assessed. The impact of obesity on eGFR trajectories and the occurrence of proteinuria was also examined.

Mortality was not accessed since only six donors died during long-term follow-up. The causes were neoplasia (*n* = 4), sudden unexplained death without autopsy (*n* = 1), and suicide (*n* = 1). None of these events could be directly attributed to donation. Given the small number of cases, meaningful subgroup or stratified analyses were not feasible.

### 2.4. Statistical Analysis

Continuous data were described using mean and standard deviation (SD) or median and interquartile range (IQR), and categorical data were expressed as numbers and percentages. Categorical data were compared using Pearson chi-square test or Fisher exact test, and continuous variables were compared with Student’s *t*-test or Mann–Whitney U test.

In order to handle the high number of missing weight data in different time points, we first approached the analysis using a multivariate mixed logistic regression, which imputed subject-specific random effects (slope defined as time in years) on an unstructured covariance matrix. The dependent variable was all obesity prevalence time-point data, and the independent variables were those considered to have a potential clinical correlation with changes in obesity status over time.

Matched pair analysis using McNemar’s test was performed to compare obesity prevalence in donors at defined time points: at donation, 1, 3, 5, and 10 years post-donation. Significance was sought comparing post-donation time points with pre-donation status. This type of analysis is more powerful than commonly utilized unpaired or independent tests it eliminates variation between samples that could be attributed to extraneous factors, given its intra-patient longitudinal comparison design. Cases with missing data were pairwise excluded. We then stratified this statistical approach considering relevant clinical factors: BMI, sex, and age. Moreover, cardiovascular risk factors prevalence (hypertension, proteinuria, dyslipidemia) was again compared at different time points, in a pairwise fashion, stratified by obesity status, and compared by McNemar’s test within each group (obese and non-obese) and by unpaired proportion test between each group (obese vs. non-obese). Similarly, mean eGFR longitudinal trends were analyzed and significance was sought by paired and unpaired *t*-test.

Statistical calculations were performed using STATA/MP, version 15.1 (Stata Corp, College Station, TX, USA). A two-sided *p*-value < 0.05 was considered statistically significant.

## 3. Results

### 3.1. Population Characteristics

The baseline characteristics of this cohort are summarized in Table 1.

Most donors (49%) had normal weight, 41% were overweight and 10% were obese. The mean age of the cohort was 47.2 ± 10.7 years, with obese donors being significantly older (50.8 ± 8.8 years, *p* = 0.009). Female donors represented 71% of the total sample.

The prevalence of dyslipidemia and hypertension increased with higher BMI, with both conditions being significantly more common in the obese group (41%, *p* < 0.001 for both). Overweight donors also had higher rates of dyslipidemia (15%, *p* < 0.001) and hypertension (19%, *p* < 0.001) compared to the normal weight group. Smoking habits varied across BMI categories, with the highest prevalence in the normal weight group (21%, *p* = 0.045). No cases of diabetes mellitus or other significant comorbidities were present, consistent with the rigorous donor selection process.

There were no significant differences between the groups regarding serum creatinine levels or urine protein–creatinine ratios. However, the pre-donation eGFR was higher in normal-weight donors (102.4 ± 15.0 mL/min/1.73 m^2^) compared to obese donors (96.3 ± 14.5 mL/min/1.73 m^2^, *p* = 0.038).

In our center, procurement of the left kidney is generally preferred because of the longer renal vein, which facilitates implantation. The left kidney was procured in 82% of cases (251 donors), with no significant difference across subgroups. Right kidney procurement was performed only in cases of anatomical variations or relative differences in split renal function.

### 3.2. Evaluation of Obesity Prevalence and Predictors

**Figure 1:**
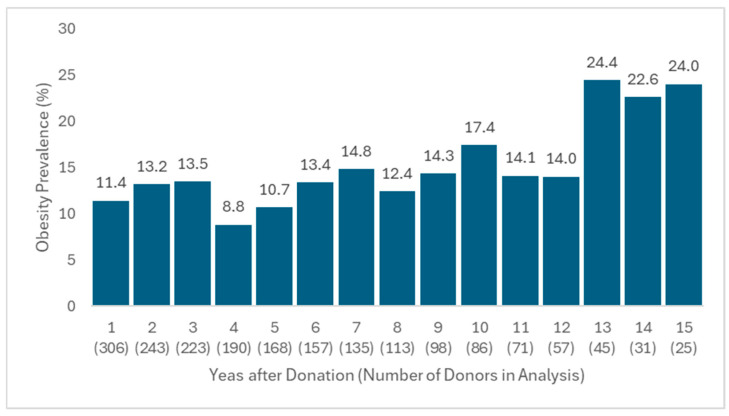
Longitudinal Obesity Prevalence in Percentage During 15 Years of Follow-up Post-Donation. Number of patients in analysis in parenthesis.

We conducted a longitudinal analysis over a 15-year follow-up period after kidney donation to evaluate obesity prevalence. During the first 9 years of follow-up, obesity prevalence ranged from 8.8% to 14.8% (Figure 1, Supplementary Table S1). Although there is variability within this period, the overall trend did not show a sustained increase. In contrast, after year 10, prevalence rose more consistently, reaching over 24.4% by year 13, but the number of donors in analysis was progressively inferior. For precise percentages, according to the year of follow-up, the Supplementary Table S1 can be consulted.

A mixed logistic regression model identified several significant predictors of post-donation obesity over the 15-year period, as demonstrated in Table 2. Age at the time of donation was inversely associated with the risk of post-donation obesity (OR 0.882, 95% CI 0.808–0.963, *p* = 0.005), suggesting that younger donors are more likely to develop obesity post-donation. As anticipated, a higher pre-donation BMI was a strong independent predictor of post-donation obesity (OR 5.324, 95% CI 3.471–8.168, *p* < 0.001), such as dyslipidemia at the time of donation (OR 6.048, 95% CI 1.065–34.348, *p* = 0.042).

### 3.3. Trends in BMI Categories

**Figure 2:**
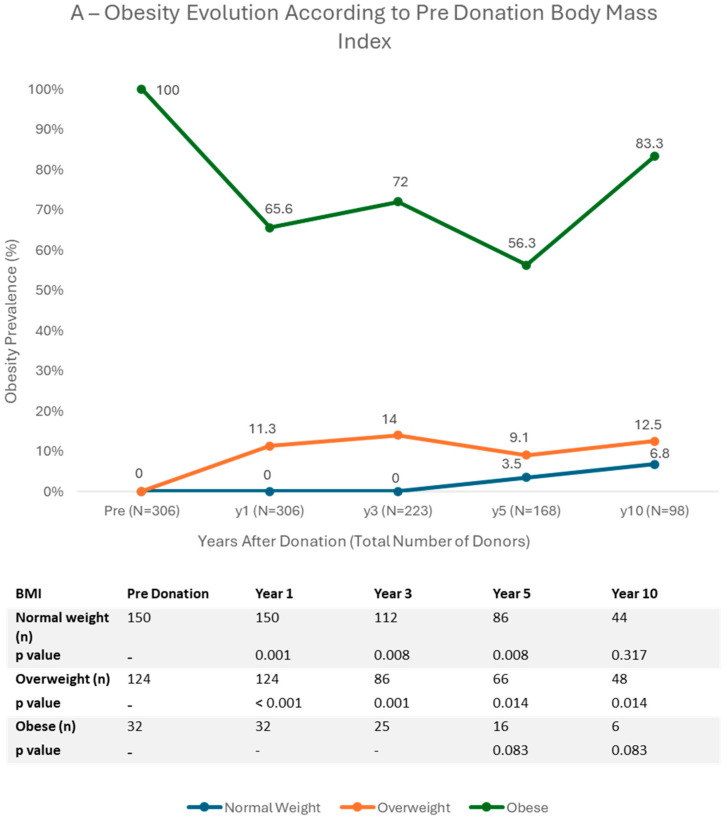

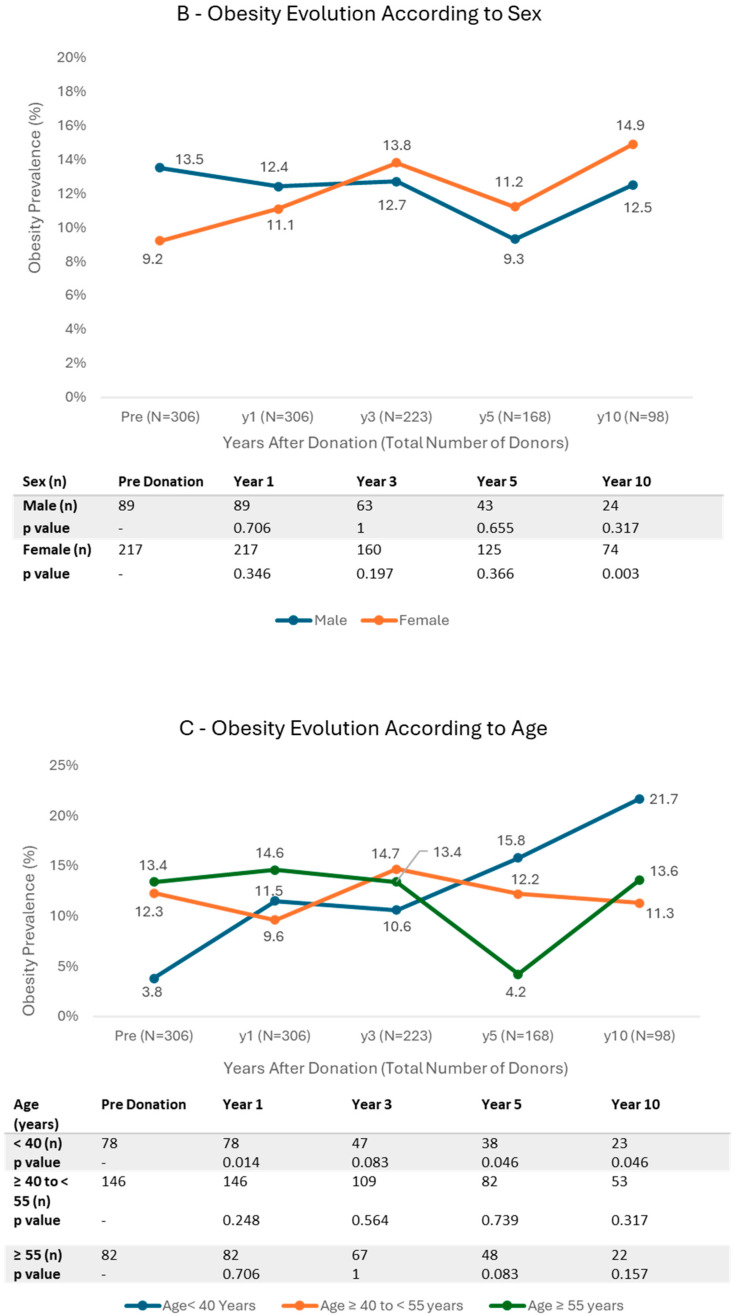
Evaluation of obesity percentage according with three different aspects: pre-donation BMI category (**A**), donor sex (**B**) and pre-donation age (**C**) using McNemar’s paired analysis in 10 years of follow up. Overweight patients (**A**) had significantly higher obesity prevalence in the follow up. Donor sex (**B**) did not have impact in obesity prevalence in the majority of the follow-up. Younger age (**C**) was associated with increased obesity prevalence during all the follow-up.

Figure 2 and Supplementary Table S2 represent McNemar’s paired analysis, which evaluates obesity evolution according with three different categories: pre-donation BMI category, donor sex, and pre-donation age.

Over a 10-year follow-up period, kidney donors exhibited varying weight trajectories depending on their initial weight status (Figure 2A). Patients classified as normal weight pre-donation demonstrated minimal progression toward obesity, with only 6.8% becoming obese by year 10 (*p* = 0.083), suggesting a relatively low risk of obesity development for those starting in a normal weight range. In contrast, overweight donors showed a steady increase in obesity rates over time, with 11.3% classified as obese by year 1 (*p* < 0.001), 14% by year 3 (*p* = 0.001), and 12.5% at year 10 (*p* = 0.014), indicating a higher risk of long-term weight gain for this group. Interestingly, donors who were initially obese experienced an early decline in obesity prevalence, dropping to 65.6% by year 1 and further to 56.3% by year 5 (*p* = 0.083). However, by year 10, the obesity rate had rebounded to 83.3% (*p* = 0.083), indicating some weight regain in later years.

The difference between sexes (Figure 2B) did not appear to be clinically relevant in relation to obesity trends across most time points. However, a statistically significant difference was observed in females at the 10-year mark, with 14.9% (*p* = 0.003) being obese, an increase from 9.2% at donation.

The data on Figure 2C suggests that younger patients, particularly those under 40, are at an increased risk of becoming obese over time, an observation that confirms the result from the mixed logistic regression analysis. At 1 year, the obesity rate rose significantly to 11.5% (*p* = 0.014), indicating an early and notable increase in this age group. This trend continued, with further significant increases at 5 years (15.8%, *p* = 0.046) and 10 years (21.7%, *p* = 0.046).

### 3.4. Cardiovascular Risk Factors and Kidney Function Trends According to BMI

**Figure 3:**
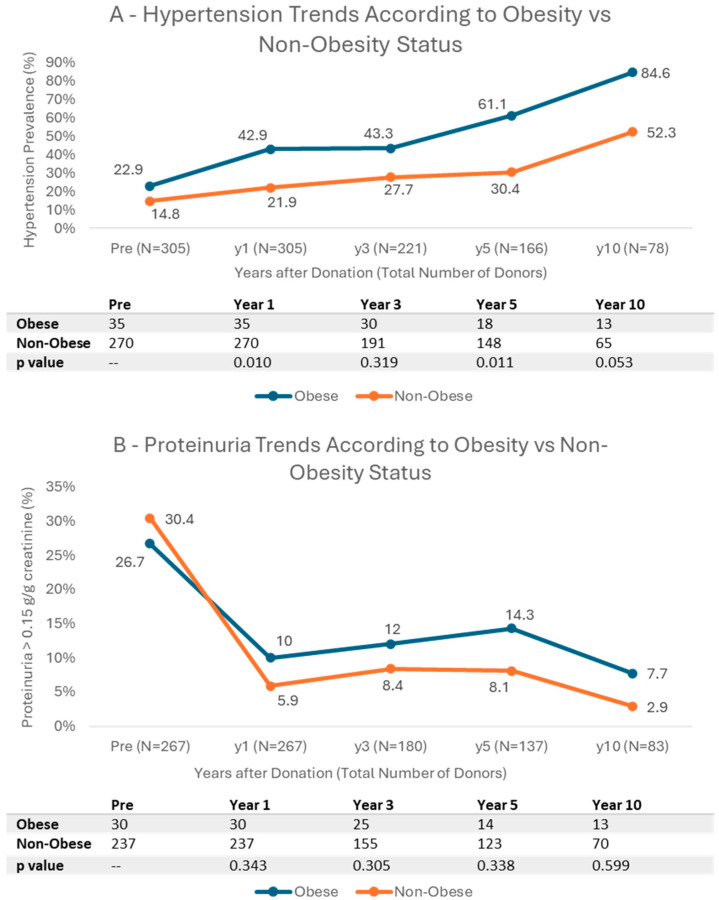

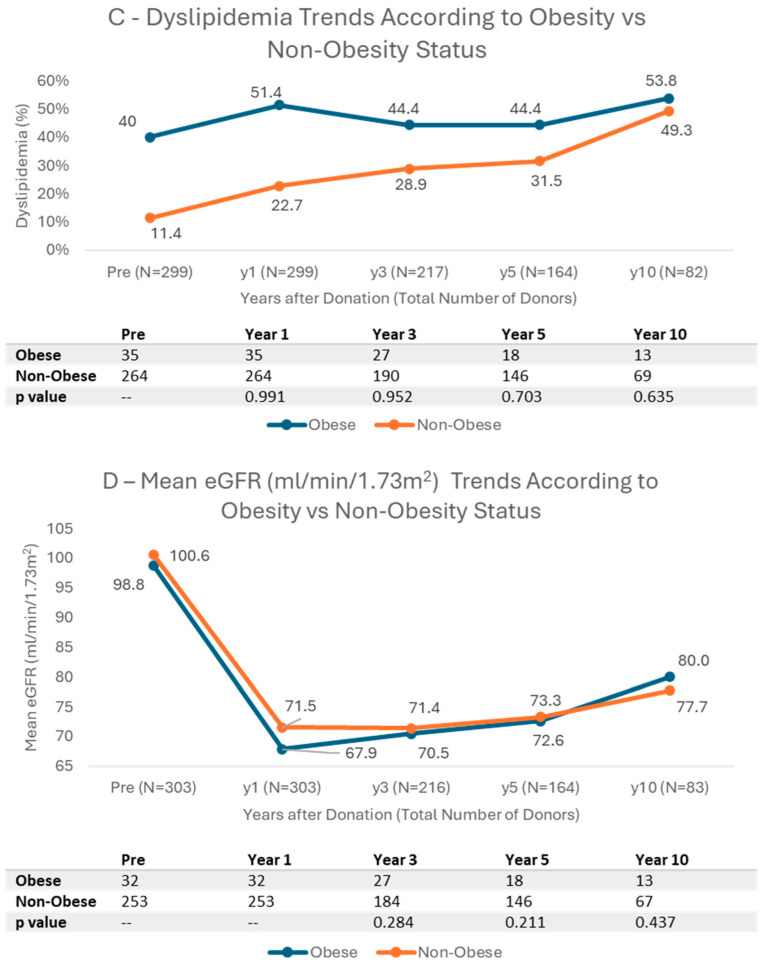
Evaluation of Hypertension (**A**), Proteinuria (**B**), Dyslipidemia (**C**), and Mean estimated glomerular filtration rate (**D**) trends according to Obese vs. Non-Obese Status in 10 years of follow-up. Hypertension (**A**) had significantly higher prevalence in the obese group. There were no differences between the obese and non-obese groups concerning proteinuria prevalence (**B**), dyslipidemia prevalence (**C**), and mean estimated glomerular filtration rate (**D**).

Hypertension prevalence increased across all groups following kidney donation (Figure 3A and Supplementary Table S3). However, a significant disparity is observed between obese and non-obese patients. At 1 year post-donation, hypertension was significantly more prevalent among obese patients (42.9%) compared to non-obese patients (21.9%, *p* = 0.010). This trend persisted at 5 years, where hypertension affected 61.1% of obese patients, in contrast to 30.4% of non-obese patients (*p* = 0.011).

No significant differences were observed between groups regarding proteinuria, dyslipidemia, or eGFR changes post-donation (Figure 3B–D and Supplementary Tables S4–S6).

The cohort consisted of a highly selected group of donors believed to be healthy, resulting in only 4 cases of diabetes, 3 in the non-obese cohort and 1 in the obese cohort. This limited the statistical power to detect significant differences for this variable. This information can be consulted in Table S7 of the Supplementary Material.

## 4. Discussion

In this study, most kidney donors (49%) had a normal weight, while 10% were classified as obese. Obese donors were generally older and more likely to have dyslipidemia and hypertension compared to those with normal weight. Before donation, normal-weight donors had better kidney function than obese donors (eGFR 102.4 ± 15.0 vs. 96.3 ± 14.5 mL/min/1.73 m^2^, *p* = 0.038).

During the first nine years, obesity prevalence fluctuated between 8.8% and 14.8%, without evidence of a sustained upward trend. This observation is further supported by the lack of statistical significance for time after donation in the multivariable mixed logistic regression. The modest increase observed toward the end of the follow-up period may reflect follow-up loss or surveillance bias, as donors with obesity or related complications may have been more likely to remain under closer medical monitoring.

The mixed logistic regression model identified three key predictors of post-donation obesity: younger age, higher BMI before donation, and dyslipidemia. These findings were reinforced by McNemar’s paired analysis, which showed that overweight donors were the group most likely to become obese after donation, particularly those under 40 years old. Normal-weight donors generally maintained their weight, while obese donors initially reduced their obesity prevalence post-donation, but this trend reversed in later years. McNemar’s paired analysis also showed no significant difference between sexes regarding obesity development.

Among comorbidities, hypertension was significantly more common in obese donors compared to non-obese donors. However, dyslipidemia, proteinuria and eGFR changes did not differ significantly between groups. Diabetes prevalence was low, with only a few cases in both groups.

### 4.1. Obesity and Age

The observed association between post-donation obesity and younger age (particularly under 40 years) is a novel finding, supported by both the mixed logistic regression model and McNemar’s paired analysis. While this relationship has not been extensively described in the literature, it may be partly explained by older donors being more prone to sarcopenia, having a greater burden of comorbidities, or showing higher adherence to dietary counseling. Importantly, BMI is only a surrogate marker of body composition and carries notable limitations. A low BMI is often regarded as favorable; however, in older donors, it may instead reflect sarcopenia rather than a truly healthy metabolic profile [34]. Sarcopenia, characterized by reduced muscle mass and strength, can mask adverse health status and complicate the interpretation of BMI. In addition, reduced muscle mass in older donors may result in lower creatinine generation, potentially leading to an overestimation of eGFR when using creatinine-based equations. Future studies should therefore incorporate complementary assessments of body composition, such as bioimpedance analysis or imaging-based techniques, to more accurately differentiate healthy leanness from sarcopenia in living kidney donors.

### 4.2. Pre Donation Body Mass Index

The influence of higher pre-donation BMI on post-donation obesity was especially evident among overweight donors, consistent with prior studies. Bugeja et al. [35] conducted a single-center study involving 151 LKD who donated between 2009 and 2017, with a median follow-up of 392 days post-donation, showing that among overweight and obese donors (BMI > 25 kg/m^2^), weight increased significantly after donation, from 86.0 ± 2.1 kg to 88.8 ± 2.7 kg (mean difference 2.3 ± 0.9 kg, *p* < 0.0001). Similarly, Punjala et al. [36] performed a retrospective analysis of 303 donors who proceeded to donate between 2012 and 2016. They reported that while some obese donors lost weight before donation (mean BMI difference of −1.32 kg/m^2^, *p* < 0.001), their weight returned to baseline levels one year after donation, with weight gain persisting at the two-year follow-up (mean BMI difference of +1.47 kg/m^2^, *p* < 0.001). These findings, in line with our results, emphasize the need for targeted and sustained weight control efforts in overweight and obese kidney donors.

### 4.3. Pre Donation Dyslipidemia

Pre-donation dyslipidemia also emerged as a significant predictor of post-donation obesity in our regression model. However, interpretation should be cautious, given the small number of donors with baseline dyslipidemia (14%), resulting in wide confidence intervals (OR 6.048, 95% CI 1.065–34.348, *p* = 0.042). Despite limited statistical power, this finding is biologically plausible, as dyslipidemia often reflects an adverse metabolic profile and poor nutritional habits predisposing to later weight gain. Nonetheless, post-donation dyslipidemia prevalence did not differ significantly between obese and non-obese groups.

### 4.4. Cardiovascular Risk

Concerning cardiovascular risk, obese donors had significantly higher rates of hypertension compared to non-obese donors, indicating that obesity was strongly associated with hypertension following donation. This aligns with Issa et al. [37], who reported an increased risk of hypertension (RR 1.93, 95% CI 1.51–2.46, *p* < 0.001) associated with post-donation weight gain. Given the well-established link between obesity and hypertension, this relationship is likely exacerbated in LKDs, where the prevalence of hypertension is elevated due to reduced kidney mass [38]. It is important though to recognize that the studies that addressed the blood pressure changes post-donation showed that the differences in quantitative value are low. For example, Kim et al. [39] found that there was a statistically significant increase in BP post-donation, but the increase in BP was from 113/75 to 116/77 mm Hg. Thiel et al. [40] suggested that donors that develop hypertension post-donation were already pre-hypertensive before donation, which we know can be the case for obese patients. Preventive measures—including regular follow-up, early treatment initiation, and low-sodium diet—should therefore be prioritized.

Diabetes prevalence was low in our cohort, but prior studies (Issa et al. [37]) found a substantially higher risk of diabetes with post-donation weight gain (RR 4.18, 95% CI 2.05–8.5, *p* < 0.0001).

### 4.5. Kidney Function and Proteinuria

Our cohort did not have any significant differences between obese and non-obese groups in terms of proteinuria or post-donation eGFR changes. This finding should be interpreted cautiously, as obesity is well established as a risk factor for eGFR decline, pro-teinuria, and ESKD in the general population. Several possible explanations may account for the absence of significant differences in our study. First, the relatively limited number of obese donors and the length of follow-up (median 6.59 years) may have reduced the statistical power to detect differences that typically emerge after longer observation periods. Second, living kidney donors undergo rigorous medical evaluation before donation, which tends to exclude individuals at highest metabolic or renal risk. Such careful selection, as suggested by Issa et al. [37], may attenuate the impact of obesity in donor cohorts compared to the general population. Importantly, our previous study [41] examining 15-year trajectories showed that normal-weight donors had significantly better early post-donation eGFR recovery than overweight and obese donors (normal weight: +0.59 mL/min/1.73 m^2^, 95% CI: +0.37 to +0.80; overweight: +0.35, 95% CI: −0.14 to +0.56; and obese: −0.18, 95% CI: −0.68 to +0.31, *p* = 0.020), but after the first five years of follow-up, those differences did not exist. This may reflect an initial reduced adaptive hyperfiltration capacity in overweight and obese individuals. Grams et al. [42,43] similarly showed that every 5 kg/m^2^ increase in BMI above 30 increases ESKD risk by 16%, and Locke et al. [21] reported more than double ESKD risk for obese compared with non-obese donors (94 vs. 40 per 10,000 over 20 years). Praga et al. [44] also observed increased proteinuria and kidney insufficiency in obese donors, albeit typically emerging a decade or more after donation. In contrast, Serrano et al. [45] and Issa et al. [37] did not observe significant long-term differences. Overall, our findings likely reflect careful donor selection and shorter follow-up, but the broader literature strongly supports obesity as a long-term renal risk factor.

### 4.6. Limitations

Several limitations must be acknowledged. Although the study included follow-up periods of up to 15 years, the median follow-up was only 6.6 years, which may be insufficient to capture late complications such as ESKD that typically emerge beyond the first decade after donation. In addition, only a relatively small number of donors were followed beyond 10 years, reducing statistical power to detect long-term differences. Another important limitation is the absence of information on BMI trajectories before donation, which prevents us from knowing whether donors intentionally modified their weight in preparation for nephrectomy. Selection bias must also be considered, as the study population comprised exclusively medically suitable living donors, a group that is healthier than the general population. This careful selection process likely reduced the observed risks and limits the applicability of our findings to broader or less healthy populations.

Additional limitations relate to unmeasured variables. We did not collect data on inflammatory biomarkers such as C Reactive Protein, IL-6, or adipokines, despite increasing evidence that chronic low-grade inflammation plays a key role in the metabolic and cardiovascular risks associated with obesity [46,47]. The cohort was predominantly female, raising concerns about sex-related generalizability, as weight gain patterns and cardiometabolic risk may differ between men and women. Ethnicity variability was also scarce, which is relevant since cultural, lifestyle, and genetic factors strongly influence weight trajectories and cardiometabolic outcomes. Furthermore, several important potential confounders were not captured, including dietary habits, physical activity, socioeconomic status, and family history of metabolic disease—all of which could significantly affect obesity development and long-term health outcomes.

Finally, this was a single-center Portuguese study, which may limit the external generalizability of our findings. Donor populations, healthcare practices, and selection criteria vary across countries and centers, potentially influencing obesity prevalence and its predictors.

Nonetheless, the main risk factors identified in our cohort—higher pre-donation BMI, younger age, and dyslipidemia—are well-recognized metabolic determinants, suggesting that similar associations are likely to be observed in other settings.

### 4.7. Clinical Implications

The acceptance of overweight or obese donors remains controversial, with guideline variability [29,43,48,49,50]. The 2015 European Renal Best Practice guidelines recommend that individuals with a body mass index (BMI) greater than 35 kg/m^2^ should not be considered for living kidney donation [49]. However, the British Transplantation Society and Kidney Disease: Improving Global Outcomes (KDIGO) guidelines suggest that each case should be evaluated individually for donors with a BMI greater than 30 kg/m^2^, without specifying an absolute cut-off value [43,46].

Given the scarcity of donor organs and the favorable results from our study—showing no increased risk of proteinuria or eGFR decline in donors who became obese after donation—we argue that overweight and obese individuals should be included as potential living kidney donors. While these findings should be interpreted with caution, as they diverge from the broader literature linking obesity with long-term renal risk [21,42,43,44], they suggest that excluding such candidates outright may be unnecessarily restrictive. Instead, the focus should be on encouraging overweight and obese donors to achieve a healthier weight before donation when feasible, and on implementing structured, post-donation protocols centered on targeted weight control, nutritional counseling, and long-term follow-up. This approach balances organ availability with donor safety and provides a practical pathway for integrating higher-BMI individuals into the donor pool without disregarding their elevated metabolic risks.

Preventive strategies should focus on targeted counseling for high-risk individuals—particularly younger donors, those with pre-donation overweight, and those with dyslipidemia—combined with sustained weight management interventions both before and after donation, as well as closer long-term follow-up. Locke et al. [21] emphasized that BMI is the only donor factor that can be modified prior to donation to reduce long-term ESRD risk. In this context, integrating key predictors such as pre-donation BMI, younger age, and dyslipidemia into a practical “Obesity Risk Score” could be a valuable tool during donor evaluation. Such a score would allow stratification of candidates according to their risk of developing post-donation obesity, enabling more tailored counseling, preventive strategies, and individualized follow-up intensity.

In addition, novel therapeutic approaches—including glucagon-like peptide-1 receptor agonists (GLP-1a) and sodium–glucose cotransporter-2 inhibitors (SGLT2i)—should be considered as adjuncts to lifestyle modification, given their emerging roles in weight management and cardiometabolic protection. Future research should explore whether combining structured lifestyle interventions with pharmacologic therapy in high-risk donors, identified through an obesity risk stratification tool, can mitigate long-term obesity-related complications after kidney donation.

## 5. Conclusions

In this 15-year follow-up of living kidney donors, post-donation obesity remained stable during the first decade but increased thereafter, partly influenced by reduced follow-up. Higher pre-donation BMI, younger age, and dyslipidemia independently predicted post-donation obesity; obesity was strongly associated with hypertension but not with differences in proteinuria or eGFR decline over the available follow-up. Given organ scarcity—and despite these favorable findings diverging from prior literature—we support including overweight and obese candidates for donation, with preference for pre-donation weight optimization and structured, post-donation weight-control and monitoring protocols to safeguard outcomes. Multicenter studies with larger, more diverse cohorts and longer follow-up are needed to validate and extend these results.

## Figures and Tables

**Table 1:** Baseline Characteristics of Kidney Donors Stratified by Pre-Donation Body Mass Index Status.

	Total	Normal Weight (BMI < 25 kg/m^2^)	Overweight (BMI 25–29.9 kg/m^2^)	Obese (BMI ≥ 30 kg/m^2^)	*p*
N (%)	306 (100%)	150 (49%)	124 (41%)	32 (10%)	-
Age (years), mean ± SD	47.2 ± 10.7	45.4 ± 11.3	48.4 ± 10.2	50.8 ± 8.8	0.009
Age (years), n (%)					
<40	78 (25)	50 (33)	25 (20)	3 (9)	0.021
40–55	146 (48)	65 (43)	63 (51)	18 (56)	
≥55	82 (27)	35 (23)	36 (29)	11 (34)	
Sex F, n (%)	217 (71)	114 (76)	83 (67)	20 (63)	0.140
BMI (kg/m^2^), mean ± SD	25.3 ± 3.4	22.5 ± 1.7	27.3 ± 1.4	31.2 ± 1.1	-
Smoking habits, n (%)	49 (16)	32 (21)	13 (10)	4 (13)	0.045
Dyslipidemia, n (%)	44 (14)	13 (9)	18 (15)	13 (41)	<0.001
Total cholesterol (mg/dL), mean ± SD	194.3 ± 37.2	188.2 ± 36.0	196.5 ± 36.2	214.3 ± 40.2	0.001
HT, n (%)	48 (16)	11 (7)	24 (19)	13 (41)	<0.001
SBP (mmHg), mean ± SD	122.5 ± 13.4	118.8 ± 12.0	125.0 ± 13.3	130.0 ± 14.3	<0.001
DBP (mmHg), mean ± SD	73.2 ± 8.7	71.6 ± 8.2	74.2 ± 8.9	76.8 ± 8.8	0.002
ProtU 0.15–0.5 g/g, n (%)	87 (28)	43 (29)	33 (27)	11 (34)	0.683
Serum creatinine (mg/dL), mean ± SD	0.75 ± 0.16	0.73 ± 0.15	0.76 ± 0.17	0.78 ± 0.15	0.115
Pre- donation eGFR (mL/min/1.73 m^2^), mean ± SD	100.3 ± 14.6	102.4 ± 15.0	98.9 ± 13.9	96.3 ± 14.5	0.038
Pre- donation eGFR (mL/min/1.73 m^2^), n (%)					0.278
<80	27 (9)	14 (9)	10 (8)	3 (9)	
80–90	47 (15)	17 (11)	22 (18)	8 (25)	
≥90	232 (76)	119 (79)	92 (74)	21 (66)	
Related donor, n (%)	104 (34)	52 (35)	40 (32)	12 (38)	0.830
Donation of left kidney	251 (82)	115 (77)	108 (87)	28 (88)	0.624

BMI: Body Mass Index; SD: standard deviation; F: female; HT: Hypertension; SBP: Systolic Blood Pressure; DBP: Diastolic Blood Pressure; ProtU: Proteinuria; eGFR: Estimated Glomerular Filtration Rate.

**Table 2:** Risk Factors at Donation for Post-Donation Obesity (Mixed Logistic Model).

	Multivariable OR (95% CI)	*p*
Time post-donation	0.985 (0.844–1.150)	0.848
Age	0.882 (0.808–0.963)	0.005
Female Sex	3.250 (0.731–14.453)	0.122
Pre-donation BMI kg/m^2^	5.324 (3.471–8.168)	<0.001
Smoking habits	0.450 (0.055–3.647)	0.454
HT	0.182 (0.030–1.094)	0.063
Dyslipidemia	6.048 (1.065–34.348)	0.042
ProtU 0.15–0.5 g/g	0.361 (0.083–1.560)	0.172
Pre-donation eGFR mL/min/1.73 m^2^	0.951 (0.899–1.006)	0.080
Related donor	0.745 (0.194–2.831)	0.668

OR: Odds Ratio; CI: Confidence Interval; BMI: Body Mass Index; HT: Hypertension; ProtU: Proteinuria (Protein–Creatinine Ratio); eGFR: Estimated Glomerular Filtration Rate.

## Data Availability

The original contributions presented in this study are included in the article/Supplementary Material. Further inquiries can be directed to the corresponding author.

## Supplementary Materials

The following supporting information can be downloaded at https://www.mdpi.com/article/10.3390/jcm14186411/s1. Table S1: Longitudinal Obesity Prevalence During 15 Years of Follow-up of Living Kidney Donors Post-Donation; Table S2: Evaluation of body mass index evolution according with three different aspects: pre donation BMI category, donor sex, and pre-donation age (McNemar’s paired analysis); Tabel S3: Hypertension Trends For a 10 Years Follow-up According to Obese and Non-Obese Status; Table S4: Proteinuria Trends For a 10 Years Follow-up According to Obese and Non-Obese Status; Table S5: Dyslipidemia Trends For a 10 Years Follow-up According to Obese and Non-Obese Status; Table S6: Estimated Glomerular Filtration Rate (eGFR) Trends For a 10 Years Follow-up According to Obese and Non-Obese Status; Table S7: Diabetes Mellitus (DM) Trends For a 10 Years Follow-up According to Obese and Non-Obese Status.

## Abbreviations

The following abbreviations are used in this manuscript:

BMI	Body Mass Index
BP	Blood Pressure
CI	Confidence Interval
CKD	Chronic Kidney Disease
eGFR	Estimated Glomerular Filtration Rate
ESKD	End-Stage Kidney Disease
HR	Hazard Ratio
IQR	Interquartile Range
KDIGO	Kidney Disease: Improving Global Outcomes
LKD	Living Kidney Donor
LKDT	Living Kidney Donor Transplantation
OR	Odds Ratio
SD	Standard Deviation
