# Supplementary material for: Prevalence of Obesity After Living Kidney Donation and Associated Risk Factors: Cardiovascular and Renal Implications
Source: J Clin Med. 2025 Sep 11;14(18):6411. doi: 10.3390/jcm14186411 (PMC12470812; doi:10.3390/jcm14186411)
Supplement: Supplementary file 1 [file jcm-14-06411-s001.zip › jcm-3811812-supplementary.pdf]

Supplementary Material

Table S1: Longitudinal Obesity Prevalence During 15 Years of Follow-up of Living Kidney Donors Post-Donation.

Year	Obese (%)
1	11.4
2	13.2
3	13.5
4	8.8
5	10.7
6	13.4
7	14.8
8	12.4
9	14.3
10	17.4
11	14.1
12	14.0
13	24.4
14	22.6
15	24.0

Table S2: Evaluation of body mass index evolution according with three different aspects: pre donation BMI category, donor sex and pre-donation age (McNemar's paired analysis).

		**Obesity trends**							
		**Pre**	**y1**	**Pre**	**y3**	**Pre**	**y5**	**Pre**	**y10**
**Overall**	N	306		223		168		98	
	N (%) obese	32 (10.5)	35 (11.4)	25 (11.2)	30 (13.5)	16 (9.5)	18 (10.7)	6 (6.1)	14 (14.3)
	Mean difference % (95% CI)	1.0 (-4.5;2.5)		2.2 (-6.5; 2.0)		1.2 (-6.4; 4.1)		8.2 (1.0-15.3)	
	P-value	0.549		0.251		0.617		**0.011**	
**Normal weight**	N	150		112		86		44	
	N (%) obese	0	0	0	0	0	3 (3.5)	0	3 (6.8)
	Mean difference % (95% CI)	0		0		3.5 (-1.6; 8.5)		6.8 (-2.9; 16.5)	
	P-value	-		-		0.083		0.083	
**Overweight**	N	124		86		66		48	
	N (%) obese	0	14 (11.3)	0	12 (14.0)	0	6 (9.1)	0	6 (12.5)
	Mean difference % (95% CI)	11.3 (4.9-17.7)		14.0 (5.5-22.4)		9.1 (0.6-17.5)		12.5 (1.1-23.9)	
	P-value	**<0.001**		**0.001**		**0.014**		**0.014**	
**Obese**	N	32		25		16		6	
	N (%) obese	32 (100)	21 (65.6)	25 (100)	18 (72.0)	16 (100)	9 (56.3)	6 (100)	5 (83.3)
	Mean difference % (95% CI)	-34.4 (-14.8; -54.0)		-28.0 (-49.6; -6.4)		-43.8 (-74.3; -13.2)		-16.7 (-63.2; 29.8)	
	P-value	**0.001**		**0.008**		**0.008**		0.317	
**Male**	N	89		63		43		24	
	N (%) obese	12 (13.5)	11 (12.4)	8 (12.7)	8 (12.7)	5 (11.6)	4 (9.3)	4 (16.7)	3 (12.5)
	Mean difference % (95% CI)	-1.1 (-8.1; 5.8)		0 (-7.8; 7.8)		-2.3 (-14.8; 10.2)		-4.2 (-16.3; 8.0)	
	P-value	0.706		1		0.655		0.317	
**Female**	N	217		160		125		74	
	N (%) obese	20 (9.2)	24 (11.1)	17 (10.6)	22 (13.8)	11 (8.8)	14 (11.2)	2 (2.7)	11 (14.9)
	Mean difference % (95% CI)	1.8 (-2.4; 6.1)		3.1 (-2.2; 8.5)		2.4 (-3.6; 8.4)		12.2 (3.4-21.0)	
	P-value	0.346		0.197		0.366		**0.003**	
**Age<40**	N	78		47		38		23	
	N (%) obese	3 (3.8)	9 (11.5)	2 (4.3)	5 (10.6)	2 (5.3)	6 (15.8)	1 (4.3)	5 (21.7)
	Mean difference % (95% CI)	7.7 (0.5-14.9)		6.4 (-2.7; 15.5)		10.5 (1.9-22.9)		17.4 (2.4-37.2)	
	P-value	**0.014**		0.083		**0.046**		**0.046**	

**Age ≥ 40 & <55**	N	146		109		82		53	
	N (%) obese	18 (12.3)	14 (9.6)	14 (12.8)	16 (14.7)	9 (11.0)	10 (12.2)	4 (7.5)	6 (11.3)
	Mean difference % (95% CI)	2.7 (-8.1; 2.6)		1.8 (-5.3; 9.0)		1.2 (-7.2; 9.6)		3.8 (-5.4; 13.0)	
	P-value	0.248		0.564		0.739		0.317	
**Age≥ 55**	N	82		67		48		22	
	N (%) obese	11 (13.4)	12 (14.6)	9 (13.4)	9 (13.4)	5 (10.4)	2 (4.2)	1 (4.5)	3 (13.6)
	Mean difference % (95% CI)	1.2 (-6.3; 8.8)		0 (-7.3; 7.3)		-6.3 (-15.2; 2.7)		9.1 (-7.5; 25.6)	
	P-value	0.706		1		0.083		0.157	

Y: year; CI: confidence interval.

Tabel S3: Hypertension Trends For a 10 Years Follow-up According to Obese and Non-Obese Status.

		Hypertension trends							
		**Pre**	**y1**	**Pre**	**y3**	**Pre**	**y5**	**Pre**	**y10**
**Overall**	N	305		221		166		78	
	N (%) HT	48 (15.7)	74 (24.3)	41 (18.6)	66 (29.9)	27 (16.3)	56 (33.7)	10 (12.8)	45 (57.7)
	Mean difference % (95% CI)	8.5 (5.1; 12.0)		11.3 (6.5-16.1)		17.5 (11.1-23.8)		44.9 (32.6-57.2)	
	P-value	**<0.001**		**<0.001**		**<0.001**		**<0.001**	
**Non-obese***	N	270		191		148		65	
	N (%) HT	40 (14.8)	59 (21.9)	33 (17.3)	53 (27.7)	23 (15.5)	45 (30.4)	8 (12.3)	34 (52.3)
	Mean difference % (95% CI)	7.0 (3.6-10.5)		10.5 (5.4-15.6)		14.9 (8.5-21.3)		40.0 (26.6-53.4)	
	P-value	**<0.001**		**<0.001**		**<0.001**		**<0.001**	
**Obese***	N	35		30		18		13	
	N (%) HT	8 (22.9)	15 (42.9)	8 (26.7)	13 (43.3)	4 (22.2)	11 (61.1)	2 (15.4)	11 (84.6)
	Mean difference % (95% CI)	20.0 (3.9-36.1)		16.7 (0.0-33.3)		38.9 (10.8-67.0)		69.2 (36.4-100)	
	P-value	**0.008**		**0.025**		**0.008**		**0.003**	
	P-value non-obese vs obese	**0.010**		0.319		**0.011**		0.053	

*Obese and non-obese status according to the latter period in each comparison

Y: year; CI: confidence interval; HT: hypertension.

Table S4: Proteinuria Trends For a 10 Years Follow-up According to Obese and Non-Obese Status.

		**Proteinuria trends**							
		**Pre**	**y1**	**Pre**	**y3**	**Pre**	**y5**	**Pre**	**y10**
**Overall**	N	267		180		137		83	
	N (%) pu>0.15	80 (30.0)	17 (6.4)	59 (32.8)	16 (8.9)	34 (24.8)	12 (8.8)	13 (15.7)	3 (3.6)
	Mean difference % (95% CI)	-23.6 (-29.9; -17.3)		-23.9 (-31.6; 16.2)		-16.1 (-25.4; 6.7)		-12.0 (-21.7; -2.4)	
	P-value	**<0.001**		**<0.001**		**0.001**		**0.008**	
**Non-obese***	N	237		155		123		70	
	N (%) pu>0.15	72 (30.4)	14 (5.9)	48 (31.0)	13 (8.4)	31 (25.2)	10 (8.1)	11 (15.7)	2 (2.9)
	Mean difference % (95% CI)	-24.5 (-31.2; -17.7)		-22.6 (-30.9; -14.2)		-17.1 (-26.8; -7.3)		-12.9 (-23.1; -2.6)	
	P-value	**<0.001**		**<0.001**		**<0.001**		**0.007**	
**Obese***	N	30		25		14		13	
	N (%) pu>0.15	8 (26.7)	3 (10.0)	11 (44.0)	3 (12.0)	3 (21.4)	2 (14.3)	2 (15.4)	1 (7.7)
	Mean difference % (95% CI)	-16.7 (-36.2; 2.9)		-32.0 (-54.3; -9.7)		-7.1 (-45.4; 31.1)		-7.7 (-41.2; 25.8)	
	P-value	0.059		**0.005**		0.655		0.564	
	P-value non-obese vs obese	0.343		0.305		0.338		0.599	

*Obese and non-obese status according to the latter period in each comparison

Y: year; pu: urinary protein- creatinine ratio; CI: confidence interval.

Table S5: Dyslipidemia Trends For a 10 Years Follow-up According to Obese and Non-Obese Status.

		Dyslipidemia trends							
		**Pre**	**y1**	**Pre**	**y3**	**Pre**	**y5**	**Pre**	**y10**
**Overall**	N	299		217		164		82	
	N (%) dyslipidemia	44 (14.7)	78 (26.1)	34 (15.7)	67 (30.9)	23 (14.0)	54 (32.9)	11 (13.4)	41 (50.0)
	Mean difference % (95% CI)	11.4 (7.3-15.4)		15.2 (9.6-20.8)		18.9 (11.6-26.2)		36.6 (24.4-48.8)	
	P-value	**<0.001**		**<0.001**		**<0.001**		**<0.001**	
**Non-obese***	N	264		190		146		69	
	N (%) dyslipidemia	30 (11.4)	60 (22.7)	26 (13.4)	55 (28.9)	19 (13.0)	46 (31.5)	8 (11.6)	34 (49.3)
	Mean difference % (95% CI)	11.4 (7.0-15.7)		15.3 (9.4-21.1)		18.5 (10.7-26.3)		37.7 (24.1-51.2)	
	P-value	**<0.001**		**<0.001**		**<0.001**		**<0.001**	
**Obese***	N	35		27		18		13	
	N (%) dyslipidemia	14 (40.0)	18 (51.4)	8 (29.6)	12 (44.4)	4 (22.2)	8 (44.4)	3 (23.1)	7 (53.8)
	Mean difference % (95% CI)	11.4 (2.0-24.8)		14.8 (-5.7; 35.4)		22.2 (2.5-47.0)		30.8 (2.0-63.6)	
	P-value	**0.046**		0.103		**0.046**		**0.046**	
	P-value non-obese vs obese	0.991		0.952		0.703		0.635	

*Obese and non-obese status according to the latter period in each comparison

Y: year; CI: confidence interval.

Table S6: Estimated Glomerular Filtration Rate (eGFR) Trends For a 10 Years Follow-up According to Obese and Non-Obese Status.

		eGFR trends							
		**Pre**	**y1**	**Pre**	**y3**	**Pre**	**y5**	**Pre**	**y10**
**Overall**	N	303		216		164		83	
	eGFR (mean±SD)	100.4±14.6	71.1±15.5	99.7±14.7	71.3±14.6	101.5±13.6	73.2±15.7	102.2±13.6	78.1±14.7
	Mean difference % (95% CI)	-29.3 (-30.7; -27.9))		-28.4 (-30.2; -26.7)		-28.3 (-30.1; -26.5)		-24.1 (-27.2; 21.0)	
	P-value	**<0.001**		**<0.001**		**<0.001**		**<0.001**	
**Non-obese***	N	268		189		146		70	
	eGFR (mean±SD)	100.6±14.5	71.5±15.5	100.2±14.5	71.4±14.1	101.7±13.2	73.3±15.1	102.1±13.4	77.7±14.6
	Mean difference % (95% CI)	-29.1 (-30.6; -27.5)		-28.8 (-30.7; 27.0)		-28.4 (-30.3; -26.6)		-24.4 (-27.4; -21.3)	
	P-value	**<0.001**		**<0.001**		**<0.001**		**<0.001**	
**Obese***	N	35		27		18		13	
	eGFR (mean±SD)	98.8±15.6	67.9±15.6	95.9±15.5	70.5±17.6	99.8±17.3	72.6±20.3	102.7±15.4	80.0±15.3
	Mean difference % (95% CI)	-31.0 (-34.9; -27.0)		-25.5 (-31.0; -20.)		-27.2 (-34.0; -20.5)		-22.7 (-35.8; -9.7)	
	P-value	**<0.001**		**<0.001**		**<0.001**		**0.003**	
	P-value non-obese vs obese	0.368		0.240		0.807		0.783	

*Obese and non-obese status according to the latter period in each comparison

eGFR: estimated glomerular filtration rate; y: year; SD: standard deviation; CI: confidence interval.

Table S7: Diabetes Mellitus (DM) Trends For a 10 Years Follow-up According to Obese and Non-Obese Status.

		DM trends							
		**Pre**	**y1**	**Pre**	**y3**	**Pre**	**y5**	**Pre**	**y10**
**Overall**	N	285		211		164		80	
	N (%) DM	0	0	0	3 (1.4)	0	3 (1.8)	0	3 (3.8)
	Mean difference % (95% CI)	0 (-0.4;0.4)		1.4 (-0.6; 3.5)		1.8 (-0.8; 4.5)		3.8 (-1.7; 9.2)	
	P-value	1		0.083		0.083		0.083	
**Non-obese***	N	253		184		146		67	
	N (%) DM;	0	0	0	2 (1.1)	0	2 (1.4)	0	3 (4.5)
	Mean difference % (95% CI)	0 (-0.4; 0.4)		1.1 (-1.0; 3.1)		1.4 (-1.2; 3.9)		4.5 (-2.0; 10.9)	
	P-value	1		0.157		0.157		0.083	
**Obese***	N	32		27		18		13	
	N (%) DM	0	0	0	1 (3.7)	0	1 (5.6)	0	0
	Mean difference % (95% CI)	0 (-3.1; 3.1)		3.7 (-7.1; 14.5)		5.6 (-10.6; 21.7)		0 (-7.7; 7.7)	
	P-value	**1**		0.317		0.317		1	
	P-value non-obese vs obese	-		0.284		0.211		0.437	

*Obese and non-obese status according to the latter period in each comparison

DM: diabetes mellitus; CI: confidence interval.
